# Two Crystal Structures of *Bombyx mori* Lipoprotein 3 - Structural Characterization of a New 30-kDa Lipoprotein Family Member

**DOI:** 10.1371/journal.pone.0061303

**Published:** 2013-04-16

**Authors:** Agnieszka J. Pietrzyk, Anna Bujacz, Jochen Mueller-Dieckmann, Malgorzata Lochynska, Mariusz Jaskolski, Grzegorz Bujacz

**Affiliations:** 1 Center for Biocrystallographic Research, Institute of Bioorganic Chemistry, Polish Academy of Sciences, Poznan, Poland; 2 Institute of Technical Biochemistry, Faculty of Biotechnology and Food Sciences, Lodz University of Technology, Lodz, Poland; 3 Biocenter Klein Flottbeck, University of Hamburg, Hamburg, Germany; 4 Institute of Natural Fibers and Medicinal Plants, Poznan, Poland; 5 Department of Crystallography, Faculty of Chemistry, A. Mickiewicz University, Poznan, Poland; University of Queensland, Australia

## Abstract

The 30-kDa family of lipoproteins from insect hemolymph has been the focus of a number of studies over the last few years. Recently, four crystal structures of *Bombyx mori* lipoprotein 7 have been determined. Here we report two crystal structures of another member of the 30-kDa lipoprotein family, *Bombyx mori* lipoprotein 3 (Bmlp3). The protein was isolated from its natural source, mulberry silkworm hemolymph. It crystallized in two different crystal forms, Bmlp3-p21 (space group *P*2_1_) and Bmlp3-c2 (space group *C*2). The crystal structures were solved by molecular replacement using the coordinates of Bmlp7 as a starting model. The crystals of Bmlp3-p21 diffracted X-rays to 2.4 Å resolution and of Bmlp3-c2 to 2.1 Å resolution. Bmlp3 has an overall fold characteristic of 30-kDa lipoproteins, with a VHS-type N-terminal domain and β-trefoil C-terminal domain. Structural comparison of Bmlp3 and Bmlp7 shows that the loops present in the C-terminal domain are flexible and participate in dimer formation. Additionally, new putative binding sites of Bmlp3 have been analyzed in detail and the electrostatic potential of the protein surface at physiological pH 7.4 conditions has been calculated. The results of these calculations are the starting point for an explanation of the recently reported cell-penetrating properties of the 30-kDa lipoproteins.

## Introduction

Mulberry silkworm, *Bombyx mori*, was domesticated by humans at least five thousand years ago. The insect is the main producer of natural silk fiber, but nowadays it also serves as a model organism for pest control studies [1] and as a factory for production of recombinant eukaryotic proteins [2]. Mulberry silkworm is the first lepidopteran for which a draft of the genomic sequence was completed in 2004, independently by two research groups [3], [4]. Based on the full genome sequence, 14,623 silkworm genes were predicted [5]. However, the number of silkworm protein structures in the Protein Data Bank (PDB) [6] is merely ∼40, showing that it is a vastly uncharted territory.

Members of the 30-kDa lipoprotein family are the most abundant protein fraction in the hemolymph of silkworm fifth instar larvae [7]. The accumulation of these proteins in the hemolymph is developmentally regulated in a stage-dependent manner at the transcriptional level [8]. The 30-kDa proteins are synthesized in the peripheral (or larval) fat body tissue and later are secreted to the hemolymph [7]. From the hemolymph, the 30-kDa proteins are translocated into the perivisceral (or pupal) fat body tissue and utilized during adult development as storage proteins [9]. They are also transported to the yolk granules in adult female moths and serve later as yolk proteins of the developing silkworm eggs [10], [11]. It is of note that silkworm was the first lepidopteran in which the 30-kDa lipoproteins were discovered [7].

According to the genomic sequence, ten genes (*Bmlp1-Bmlp10*) of 30-kDa lipoproteins were predicted. Alignment of their deduced amino acid sequences as well as their phylogenetic analysis indicated that these proteins could be divided into three subfamilies. Most likely, all ten 30-kDa lipoprotein genes have a common ancestor that underwent differentiation and divergent evolution [12]. Moreover, differences in amino acid sequences are also observed between 30-kDa proteins from different silkworm strains [13]. Also, analysis of expressed sequence tags (ESTs) derived from several silkworm tissues indicated that the level of expression of the 30-kDa proteins varies. The more ESTs of a particular protein are present in the genome, the higher the expression level of that protein. The most abundant proteins from the 30-kDa lipoprotein family in the silkworm body are Bmlp3 and Bmlp7, with EST numbers of 607 and 864, respectively, whereas only a few ESTs were found for Bmlp6 (15 ESTs) and Bmlp8 (12 ESTs) [12]. Recently, a more comprehensive genomic analysis of the lipoprotein family [14] estimated the number of typical silkworm lipoproteins at 24 (*Bmlp1-Bmlp24*). Moreover, two additional groups of 30-kDa lipoproteins were established, namely serine/threonine-rich lipoproteins and ENF peptide-binding proteins (ENF peptides are a class of multifunctional cytokines). Finally, members of 30-kDa lipoprotein family have been found in a number of other species of the *Lepidoptera* order, e.g. *Samia cynthia ricini, Antheraea assama* or *Antheraea mylitta* [14]. Before this genomic analysis was completed, only microvitellogenin isolated from *Manduca sexta* had been reported to be homologous to silkworm 30-kDa lipoproteins [15].

**Figure 1:**
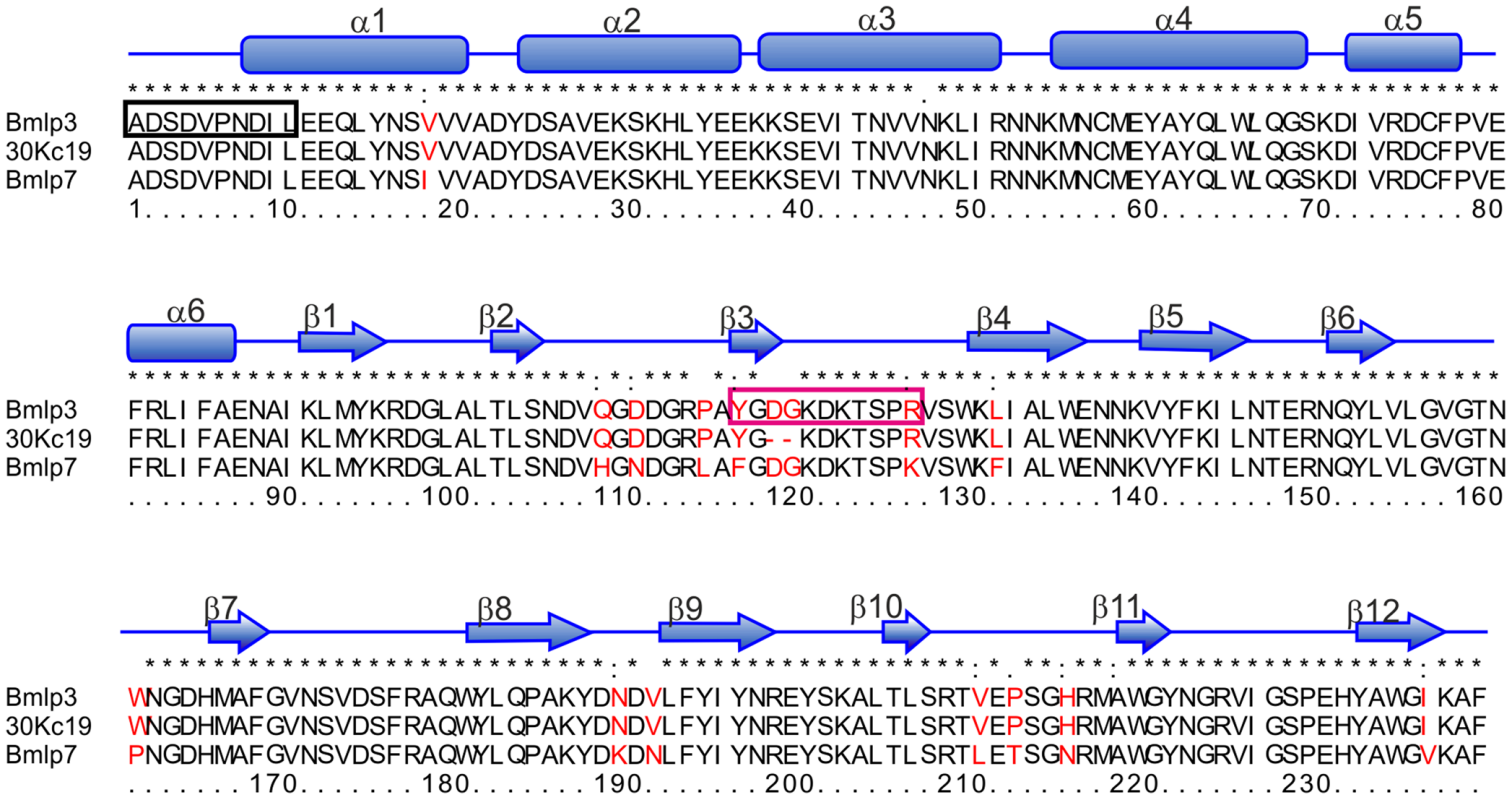
Sequence alignment of Bmlp3, Bmlp7 and 30Kc19. The amino acid sequence alignment of Bmlp3 (Silkworm Knowledgebase accession code: Bmb035159), Bmlp7 and 30Kc19 (UniProt accession codes: E5EVW2 and P09336, respectively) was calculated in ClustalW (http://www.ebi.ac.uk/Tools/msa/clustalw2/). All differences between Bmlp3, Bmlp7 and 30Kc19 are in red lettering. The N-terminal sequence established by Edman degradation is shown in a black box. The magenta box indicates the sequence fragment of Bmlp3 that might be responsible for cell-penetrating properties. The secondary structure elements are assigned as α-helices (cylinders) and β-strands (arrows).

The 30-kDa lipoproteins are an interesting group of proteins. Members of this family are involved in the immune response to fungal infections [16], [17] and our previous studies revealed that one of the 30-kDa lipoproteins, Bmlp7, might be involved in silkworm detoxification mechanisms related to heavy metal pollution [18]. The 30-kDa lipoproteins also have antiapoptotic properties: The addition of hemolymph to insect or mammalian cell cultures inhibited the apoptosis of the cells [9], [19], [20]. Recently, cell-penetrating properties of a member of the 30-kDa lipoprotein family, 30Kc19, have been reported. 30Kc19 is able to penetrate into various types of cells and localize into the mitochondria and cytoplasm. It can also deliver to those compartments various cargo, for instance the green fluorescence protein. In addition, *in vivo* tests showed that 30Kc19 injected into mice is detected in lung, kidney and liver cells [21]. 30Kc19 is an isoform of *Bombyx mori* lipoprotein 3 (Bmlp3), the protein described in this work. The amino acid sequences of the two proteins are almost identical (Fig. 1), the main difference being a two-residue insertion (_117_Gly–AspGly–Lys_120_) in what turns out to be a loop in the Bmlp3 structure.

In summary, the 30-kDa lipoproteins are very abundant in the silkworm body and are involved in a number of important biological processes. To date, only four 3D structures of insect 30-kDa proteins have been deposited in the PDB, and all of them correspond to Bmlp7. Here we present the first crystal structures of Bmlp3, which is another member of the 30-kDa lipoprotein family in *B. mori*. Our study reveals potential new binding sites in addition to those proposed earlier [22] and establishes a reliable picture of the electrostatic potential on the protein surface, which is of key importance for the understanding of its intermolecular interactions. Finally, the question of Bmlp3 dimerization is also addressed.

## Materials and Methods

### Hemolymph Collection, Protein Purification and Crystallization

*B. mori* hemolymph was collected from fifth instar larvae as described previously [23]. Bmlp3 was isolated from the hemolymph using a four-step purification protocol. The first two steps have been already described [23]. Briefly, initial protein separation was carried out using size exclusion chromatography with a Superdex 200 prep grade column (XK 16/100, Amersham Biosciences). The collected fractions containing the 30-kDa proteins were concentrated and applied on a Q Sepharose column (XK 16/10, Amersham Biosciences). Stepwise-elution ion exchange chromatography allowed further separation. Bmlp3 was eluted with 90 mM NaCl. The same fraction contained also juvenile hormone binding protein (BmJHBP). The two proteins were separated in the third purification step, hydrophobic interaction chromatography, carried out on a HiTrap Phenyl Sepharose™ HP column (Amersham Biosciences) equilibrated with 1.0 M ammonium sulfate, 10 mM Tris, pH 7.3. A linear elution gradient was applied and Bmlp3 was eluted with 0.8 M ammonium sulfate. The concentrated protein sample was desalted on a HiTrap Desalting column (Amersham Biosciences) equilibrated with 10 mM Tris, pH 7.3. The collected protein fraction of Bmlp3 was concentrated to 10 mg/ml.

Crystals of the first crystal form (Bmlp3-p21) were grown at 20°C using the hanging drop vapor diffusion method and 0.1 M CaCl_2_, 0.1 M Bis-Tris, pH 6.5, and 30% PEG MME 550 as the precipitating buffer. The crystals were tiny plates and grew in clusters. Optimization of the crystallization conditions was carried out in sitting drops using the High Throughput Crystallization (HTX) Facility at the EMBL Hamburg, Germany. The second crystal form (Bmlp3-c2) was grown using 2.0 M ammonium sulfate and 0.1 M ammonium iodide, pH 5.5, as the precipitating buffer. Big enough crystals (0.15×0.1×0.05 mm) were obtained using hanging drops at 20°C.

### Data Collection and Processing

X-Ray diffraction data for both crystal forms were collected at beamline 14.1 of the BESSY synchrotron, Berlin, Germany [24], using a MAR 225 CCD detector. The diffraction images were recorded at 100 K for one single crystal in each case using the rotation method with an oscillation of 0.5° (Bmlp3-p21) or 0.4° (Bmlp3-c2). Additional cryprotectants were not used for Bmlp3-p21 due to the presence of PEG MME 550 in the crystallization solution. A solution of 70% Tacsimate, pH 5.5 was used as a cryoprotectant for Bmlp3-c2. All crystals were mounted in nylon loops and vitrified in a stream of cold nitrogen gas. The collected images were indexed, integrated and scaled using XDS [25]. The resolution of both data sets is similar, 2.4 Å for Bmlp3-p21 and 2.1 Å for Bmlp3-c2. In the latter case, reflections from the area of the 2.2 Å ice ring were not included. Data collection and processing statistics are presented in Table 1.

**Table 1:** Data collection and refinement statistics.

	Bmlp3-p21	Bmlp3-c2
Space group	*P*2_1_	*C*2
Unit cell parameters:		
a, b, c, β [Å, °]	56.9, 124.4, 67.9, 114.8	154.1, 34.5, 93.1, 97.9
Protein molecules/ASU	4	2
V_M_ [Å^3^/Da]	2.01	2.25
Solvent content [%]	38.7	45.7
**X-ray diffraction data collection**		
Temperature [K]	100	100
Radiation source	BESSY, BL114.1	BESSY, BL114.1
Wavelength [Å]	0.918	0.918
Detector	MAR CCD 225	MAR CCD 225
Rotation range per image [°]	0.5	0.4
Total rotation range [°]	180	180
Exposure time per image [s]	20.0	17.0
Resolution [Å]	34.4–2.36 (2.46-2.36)^a^	38.1–2.10 (2.20-2.10)
Intensities measured	133 505	89 568
Unique reflections	35 189	26 553
R_merge_ b [%]	8.2 (63.6)	6.0 (49.6)
Redundancy	3.8 (3.8)	3.7 (2.7)
<I/σI>	15.7 (2.7)	15.9 (2.6)
Completeness [%]	99.5 (99.7)	91.3 (86.3)
**Refinement statistics**		
Resolution range [Å]	34.4–2.36	38.1–2.10
Reflections, total/test	35 189/1106	26 553/1325
R_work_/R_free_ c [%]	17.3/25.9	21.6/27.1
No. of protein atoms	7 817	3 878
No. of solvent atoms	220	124
R.m.s. deviations from ideal		
Bond lengths (Å)	0.015	0.019
Bond angles (°)	1.71	1.81
Average *B* factor [Å^2^]	27.7	34.8
Ramachandran statistics [%]		
-favored residues	90.7	91.7
-outliers	0.1	0.0
PDB code	4IY8	4IY9

a Values in parentheses are for the highest resolution shell.

b *R_merge_* = ∑_h_∑_j_ | I_hj_ - <I_h_> |/∑_h_∑_j_ I_hj_, where I_hj_ is the intensity of observation j of reflection h.

c *R_work_* = ∑_h_ | | F_o_| - | F_c_| |/∑_h_ | F_o_| for all reflections, where F_o_ and F_c_ are the observed and calculated structure factors, respectively. *R_free_* is calculated analogously for the test reflections, randomly selected and excluded from the refinement.

### Structure Determination and Refinement

The structure of Bmlp3-p21 was determined by molecular replacement (MR) using Phaser-MR [26] and the coordinates of chain A of Bmlp7 (PDB: 4EFP) [18] as a starting model. The structure of Bmlp3-c2 was solved by MR using MolRep [27] and the coordinates of chain A of Bmlp3-p21. The final models were completed after several cycles of manual rebuilding in COOT [28], [29] interspersed with restrained structure-factor refinement in PHENIX [30] or REFMAC5 [31] with the inclusion of TLS parameters [32]. Water molecules were added in COOT during the iterative cycles of rebuilding and refinement. The refinement statistics are summarized in Table 1.

### N-terminal Sequencing Analysis

Preparation of a Bmlp3 sample for Edman degradation included gel electrophoresis separation according to Schaegger and Jagow [33] followed by protein transfer to a PVDF Immobilon membrane, PSQ 0.22 µm (Millipore). The single protein band corresponding to the molecular weight of 28 kDa was cut and subjected to Edman degradation cycles, performed using a fully automated Procise 491 (Applied Biosystems) sequencer.

## Results and Discussion

### Overall Structure of Bmlp3 and Comparison with Bmlp7

The silkworm hemolymph Bmlp3 protein used in this study was isolated from its natural source as an unknown protein. Identification of the protein, purified using a four-step purification protocol, was carried out by N-terminal sequencing. The amino acid sequence of ten N-terminal residues (_1_ADSDVPNDIL_10_) indicated that the protein belongs to the 30-kDa lipoprotein family. The final identification of the protein was completed after its crystal structure solution according to electron density maps.

Bmlp3 crystallized as two polymorphic modifications. All Bmlp3 crystals are monoclinic but belong to different space groups, *P*2_1_ (Bmlp3-p21) and *C*2 (Bmlp3-c2). Both crystal structures were solved by molecular replacement. The starting model for Bmlp3-p21 was the structure of Bmlp7 and the molecular replacement procedure identified four protein chains in the asymmetric unit. During the refinement, the protein was identified as Bmlp3 in the Silkworm Knowledgebase (http://silkdb.genomics.org.cn/silkworm/index.jsp; accession code: Bmb035159). This sequence is entirely consistent with the sequence of both Bmlp3 crystal structures. Almost the same amino acid sequence could be downloaded from UniProt (http://www.uniprot.org) as corresponding to a low molecular mass 30-kDa lipoprotein 19G1 (accession code: C7A8A3). The only difference between Bmb035159 and C7A8A3 are positions 47 (N/S) and 218 (A/S). After the manuscript had been submitted for publication, a new entry appeared in UniProt (accession code: H9J4F6) and this sequence is entirely consistent with Bmb035159 and the present crystallographic models.

The crystal structure of the second crystal form (Bmlp3-c2) was solved using a model (Bmlp3-p21) with the correct amino acid sequence. There are two protein molecules in the asymmetric unit of the Bmlp3-c2 crystal. The crystal structures were refined to final R/R_free_ factors of 17.3/25.9% (Bmlp3-p21) and 21.6/27.1% (Bmlp3-c2).

**Figure 2:**
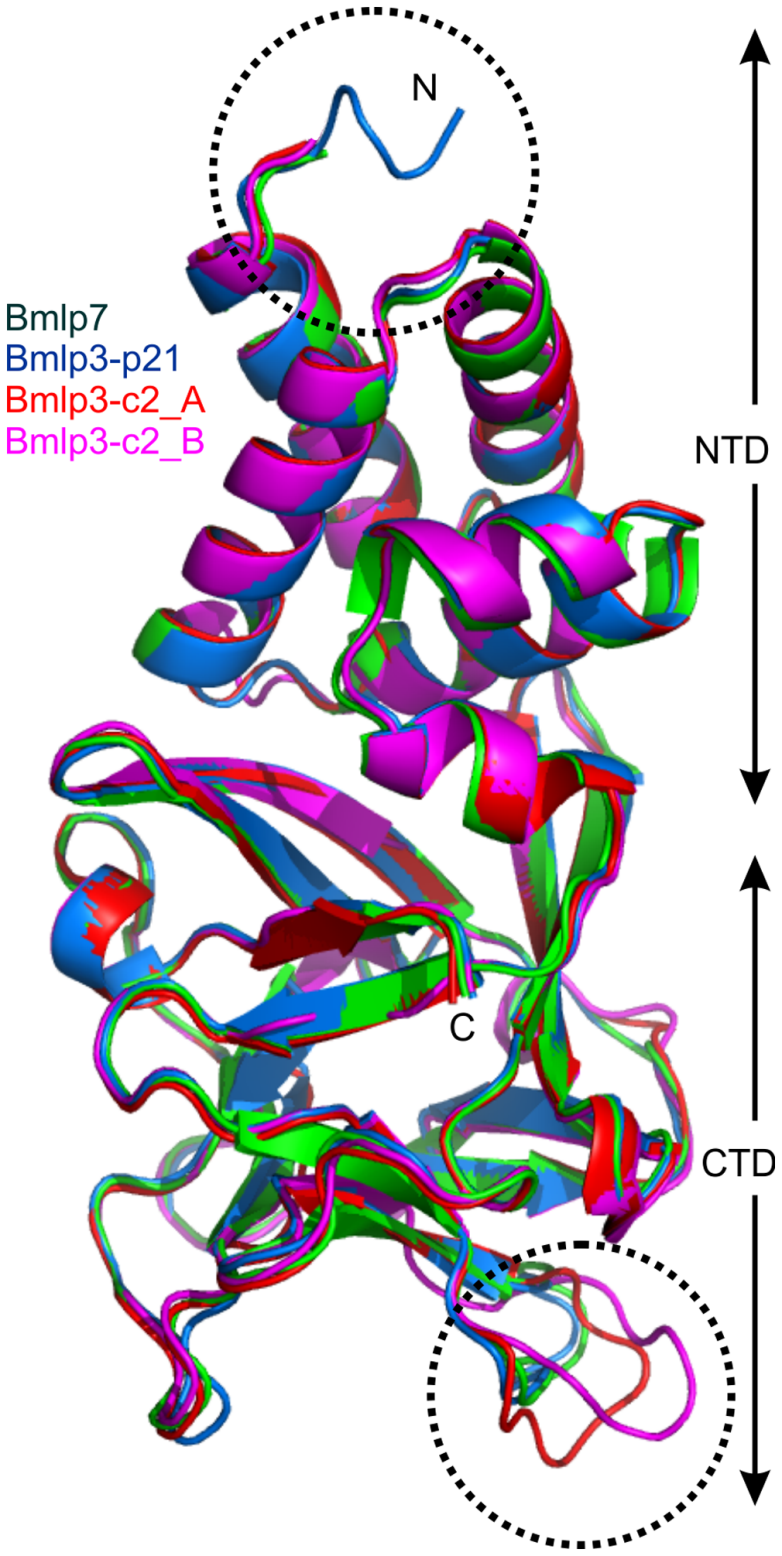
Structural comparison of Bmlp3-p21, Bmlp3-c2 and Bmlp7. The structures of Bmlp3-p21 (chain A), Bmlp3-c2 (chains A and B) and Bmlp7 (chain A) were Cα-superposed using COOT [28], [29]. The main deviations are found in the CTD domain, in loop conformation. The most flexible loop and the N-terminus are encircled.

In overall architecture, Bmlp3 has a fold characteristic of the 30-kDa lipoprotein family (Fig. 2). According to the Pfam database (http://pfam.sanger.ac.uk) all these proteins are classified as Lipoprotein_11 family (accession code: PF03260). The fold of silkworm 30-kDa lipoproteins is unique among insect lipoproteins and even among the *Lepidoptera* order. The other important insect protein, without known structure, that could possibly share the same folding canon is microvitellogenin from *Manduca sexta*. The amino acid sequence of microvitellogenin is homologous to the amino acid sequences of the 30-kDa lipoproteins [15]. For instance, the amino acid sequence alignment of microvitellogenin and Bmlp3 indicates 71% similar and 50% identical residues.

The Bmlp3 molecule consists of a helical N-terminal domain (NTD, residues 1–87) and a β-trefoil C-terminal domain (CTD, residues 88–239). The NTD consists of six helices forming a right-handed superhelix and is classified as a VHS domain [34]. The overall 30-kDa lipoprotein fold could be therefore described as a combination of VHS and β-trefoil domains. A detailed structural comparison of the lepidopteran NTD and CTD domains with classic VHS and β-trefoil domains, respectively, based on the available PDB data has been presented before [18], [22].

**Figure 3:**
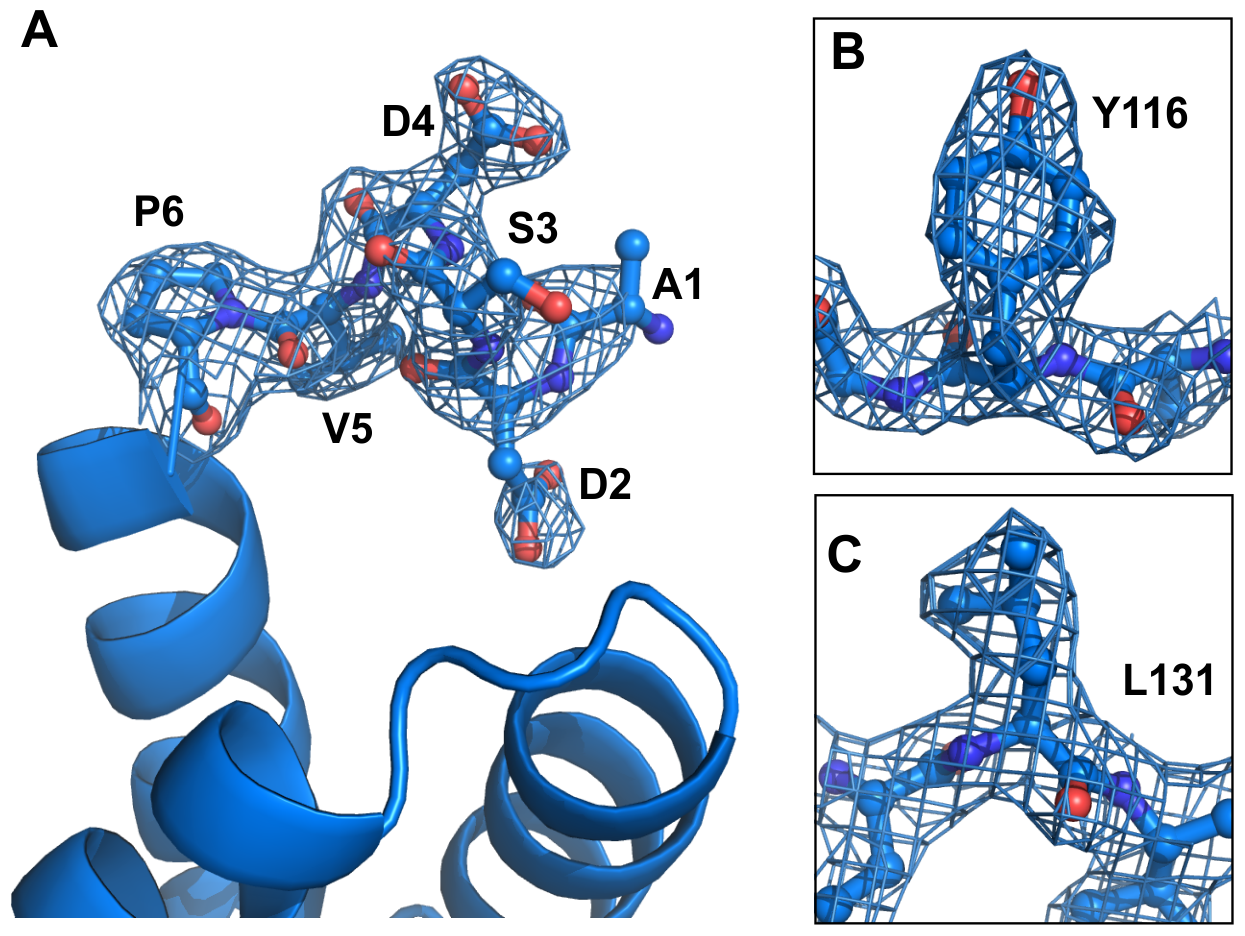
Electron density maps of Bmlp3-p21. Bmlp3-p21 is the first structure of a member of the 30-kDa lipoprotein family with complete sequence included in the model. All amino acids from the N-terminus (A) to the C-terminus were clearly identified in the electron density maps. Moreover, the quality of the electron density maps allowed for the definite identification of the protein. In most cases, the electron density of the side chains was very clear, as illustrated for Tyr116 (B) and Leu131 (C). The 2Fo-Fc maps are displayed in blue at the 1.0σ level.

The amino acid sequences of Bmlp3 and Bmlp7 (Fig. 1) contain 97% similar and 94% identical residues. Therefore, a high degree of structural similarity between these proteins is also observed. As mentioned above, all 30-kDa lipoprotein genes are likely to have a common ancestor. The r.m.s.d. values for Cα superpositions of Bmlp7 chain A (PDB: 4EFP) [18] with chain A of Bmlp3-p21 and chains A and B of Bmlp3-c2 are 0.48, 0.49 and 0.71 Å, respectively. Nevertheless, both Bmlp3 structures provide new structural information. The first important aspect is that each protein molecule traced in the Bmlp3-p21 structure contains all residues of the mature protein; it presents, therefore, the first complete model of a 30-kDa lipoprotein. The electron density was clearly defined for all N-terminal residues (Fig. 3A), which form a single α-helical turn. The Bmlp3-c2 model contains residues 5–239 in each chain.

Interestingly, the chemical modifications, such as 7-hydroxytryptopahn at position 180 (also 180 in Bmlp3) reported for Bmlp7 [18], are not present in the Bmlp3 structures. Furthermore, the conformation of several loops in the CTD in Bmlp3 differs from the conformation of these loops in Bmlp7. In particular, the loop containing residues 106–116 is very flexible (Fig. 2) and is likely to participate in a number of interactions, as explained below.

Although there are not many differences in the amino acid sequence between Bmlp3 and Bmlp7, they might be quite meaningful. It is of note that several unique amino acid positions in Bmlp3 were of key importance in the crystallographic identification of the protein. For instance, Bmlp3 has a tyrosine residue at position 116 and leucine at 131 (Fig. 3B,C), whereas in Bmlp7 these positions are occupied by phenylalanine residues. The differences in the amino acid sequences of the two proteins are sufficient to alter the pI in a noticeable way (Table 2) and this might influence their biophysical and biological properties. Our previous studies suggested that Bmlp7 might participate in silkworm detoxification mechanisms related to heavy metal pollution due to the protein’s ability to coordinate cadmium cations [18]. In order to check if Bmlp3 is capable of cadmium binding as well, several crystallization trials were performed. Specifically, cadmium chloride was added to the crystallization solutions of both crystal forms at concentrations from 0.1 to 0.5 mM. In the presence of Cd^2+^ the protein crystallized only in the form of tiny microcrystals. This suggests that the affinity of Bmlp3 for cadmium is probably lower than that of Bmlp7.

**Table 2:** Biophysical parameters for the Bmlp3 and Bmlp7 proteins calculated using ProtParam (http://web.expasy.org/protparam/).

Protein	Molecular mass [kDa]	pI	ε_280_ [M^−1^cm^−1^]
Bmlp3	27.57	6.14	62340
Bmlp7	27.53	7.11	55350

### Crystal Packing and Protein-protein Interactions

**Figure 4:**
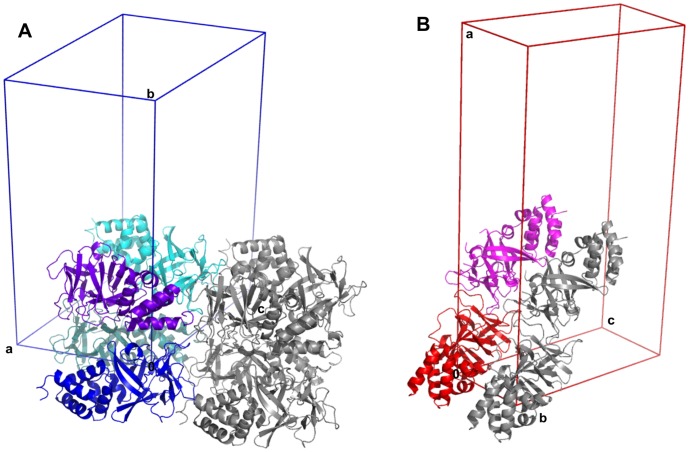
Crystal packing of Bmlp3-p21 and Bmlp3-c2. Four protein molecules (shades of blue) are present in the asymmetric unit of Bmlp3-p21 (A), whereas the asymmetric unit of Bmlp3-c2 (B) contains a dimeric assembly (red and pink). Symmetry-related molecules are shown in gray.

**Figure 5:**
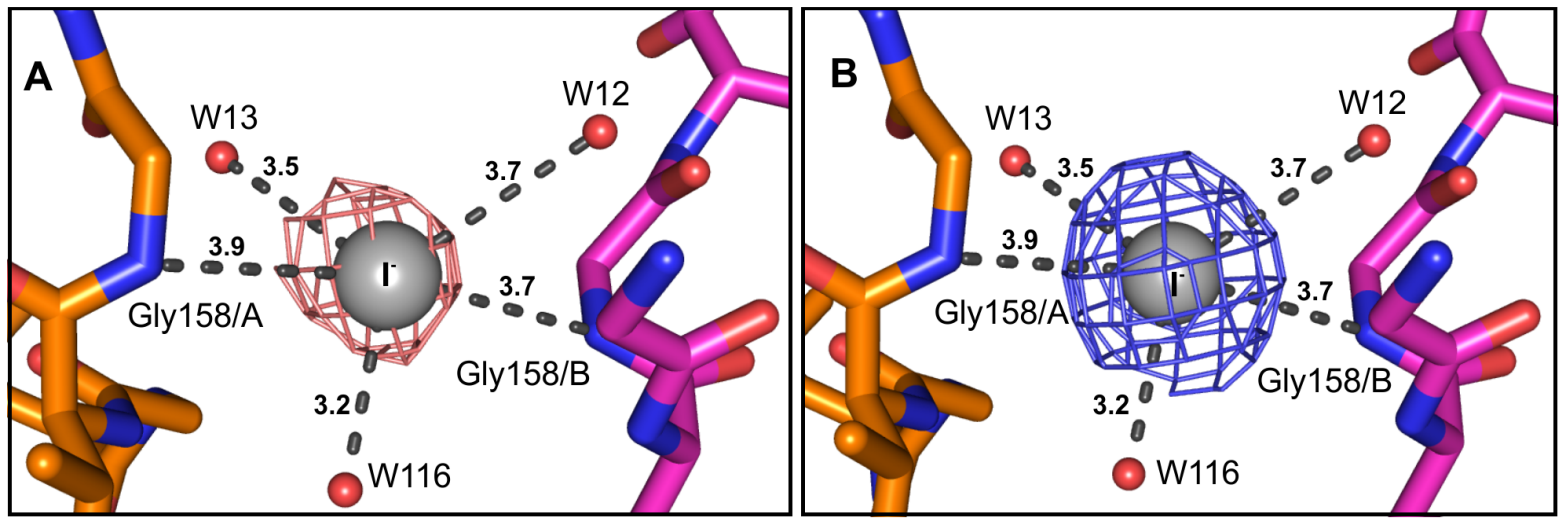
The iodide ion binding site in Bmlp3-c2. An iodide anion from the crystallization solution is present between chains A (orange) and B (magenta) of Bmlp3-c2. The N…I and O…I distances are in Å. The anomalous (A) and the 2Fo-Fc (B) maps are displayed at the iodide site at 5.0σ and at 1.0σ, respectively.

The asymmetric unit of Bmlp3-p21 contains four monomeric protein molecules (Fig. 4A), whereas in the asymmetric unit of Bmlp3-c2 two protein molecules form a dimer (Fig. 4B). The arrangement of the protein molecules differs significantly in the two crystal forms. In the Bmlp3-p21 structure, the NTD and CTD fragments are arranged alternately in a head-to-tail fashion, so that residues of the NTD have contacts with residues of the CTD. The dimer present in the Bmlp3-c2 structure is formed through interactions between the CTD domains in a tail-to-tail arrangement. The intermolecular interactions were analyzed using the PISA server [35]. Although the protein molecules in Bmlp3-p21 are packed more tightly than in Bmlp3-c2 and the solvent content is lower, the results from PISA indicate that in the Bmlp3-p21 structure there are no specific interactions leading to a stable quaternary structure. The results of PISA analysis of the Bmlp3-c2 structure suggest that the dimer present in the asymmetric unit could be also stable in solution. The total contact surface area for the crystallographic dimer is 1620 Å^2^ as calculated by PISA or 1836 Å^2^ as calculated in Areaimol from the CCP4 suite [36]. The interaction interface between the CTDs of protein molecules A and B is formed mainly by amino acid residues in loops β2–β3 and β6–β7. The space between the protein chains is filled with a number of water molecules and the protein-protein interactions are hydrophilic. The conformation of the loop containing residues 106–116 promotes these interactions. In molecule A this loop is shifted by about 5 Å compared to the same loop in Bmlp3–p21, whereas in molecule B this shift is about twice as large and the loop is bent (Fig. 2). Such an arrangement of loop 106–116 in both protein molecules stabilizes the dimer. The central part of the dimerization interface is occupied by an iodide anion from the crystallization solution. The iodide anion is located on the non-crystallographic two-fold axis. To confirm the identity of the iodide anion, the data were reprocessed with separation of the Bijvoet pairs and an anomalous difference map was generated. The diffraction data were collected at λ = 0.918 Å where the f’” anomalous correction of iodine is 2.9e. The anomalous peak at the iodide site was visible at 7.0σ. The iodide anion forms hydrogen bonds with three water molecules, with I…O distances ranging from 3.2 to 3.7 Å, and with two nitrogen atoms of the protein backbone (Gly158A/B) with the N…I distances of 3.7 and 3.9 Å (Fig. 5).

The two crystal forms were obtained using different crystallization conditions. The crystals of Bmlp3-p21 were obtained with PEG MME 550 as the main precipitant, whereas ammonium sulfate was the precipitant in the Bmlp3-c2 case. pH was also different, 6.5 and 5.5, respectively. It has been established that the pH of silkworm hemolymph is 6.5 and it decreases near the membrane surface of the silkworm cells [37]. The pH conditions of our Bmlp3 crystallization experiments reflect this physiological situation. Our dynamic light scattering (DLS) experiments (not shown) suggested that the protein is monomeric in solutions of pH 5.5 and 6.5. However, it might be speculated that while Bmlp3 is monomeric in the hemolymph milieu, it could dimerize when translocated to the pupal fat body, in a process triggered by an unknown factor, e.g. increased salt concentration. Nevertheless, this interesting issue requires further research. In our opinion the best way to prove or disprove this speculative hypothesis would be to carry out comprehensive *in vivo* studies.

### Potential Binding Sites

**Figure 6:**
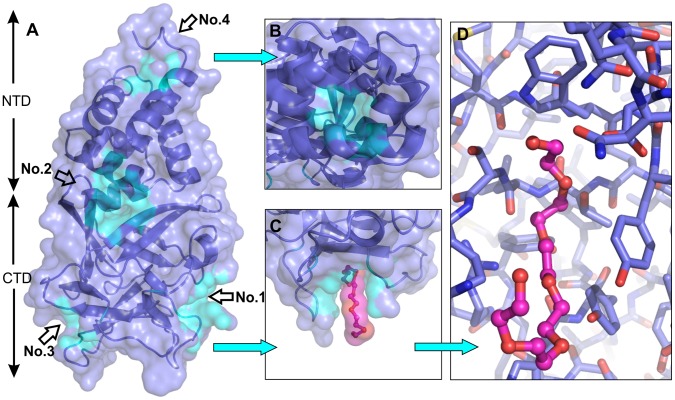
Potential Bmlp3 binding sites. Molecular cavities as putative binding sites of Bmlp3 (A) are marked by No. 1–4, as in Table 3. Cavity No. 4 (view at the top of the protein molecule, B) is the only pocket formed entirely in the NTD. Cavity No. 1 (C) has an elongated shape and might be a binding site for docking of a fatty acid chain, as well as of an oligosaccharide. A fragment of a PEG molecule from the crystallization solution is bound in cavity No. 1 of chains A and D of Bmlp3-p21 (D).

Chain A of Bmlp3-p21 was analyzed for potential binding cavities using the CASTp [38] and metaPocket 2.0 [39], [40] servers. Bmlp3-p21 was chosen for this analysis because of the absence of meaningful quaternary protein-protein interactions. Two putative binding cavities, one for a lipid and one for a saccharide ligand, were described for Bmlp7 [22]. The goal of our search was not limited to lipid- or sugar-binding sites, but targeted potential binding pockets also for other, unknown ligands. The hemolymph is a complex fluid containing many different chemical compounds [41], thus we speculate that the 30-kDa lipoproteins might be involved in binding and transport of a number of them. The result of our search are four potential binding cavities (Fig. 6A) with high scores in both CASTp and metaPocket. Table 3 summarizes the main characteristics of these cavities. Only one of them (No. 1) corresponds to those found by Yang et al. [22].

**Table 3:** Potential binding sites of the Bmlp3 protein.

Cavity number	Volume [Å^3^]	Location in the structure	Residues forming the cavity
1	399	CTD	Y94, R96, N160, N162, D164, T205, S206, R207, T208, W219, Y221, Y232
2	208	NTD-CTD	D21, D23, M55, N56, M58, E59, N138, K139, V140, Y141, F142, Y181, L182
3	110	CTD	D110, D111, V154, F168, V170, R177
4	60^a^	NTD	I9, L10, Q13, L14, K29, L33

a The volume of cavity No. 4 was estimated to be 60 Å^3^, however, it might be larger than predicted upon even a slight movement of the helices in the NTD.

Two putative binding pockets were found in the CTD domain. The largest one (No. 1 in Table 3) is homologous to a site identified by Yang et al. [22] in Bmlp7 and postulated to be a putative sugar binding site. It is formed by residues from loops β1-β2 and β6-β7, and by two residues in the β11 strand. The cavity is lined with three conserved tyrosine side chains (Y94, Y221, Y232) which could be excellent sugar binding residues. In the Bmlp3-p21 structure this cavity is occupied by a fragment of polyethylene glycol (PEG) from the crystallization solution (Fig. 6C,D). Through its ether/alcohol character, PEG is a relatively good mimic of a carbohydrate moiety. As another possibility, cavity No. 1 could be a binding site for fatty acids. It has an elongated shape and several residues (for example, the aromatic rings of Y221, Y232) could form hydrophobic interactions with the aliphatic chain of a fatty acid molecule. The cavity volume, estimated at 399 Å^3^, should be sufficient for binding of one molecule of palmitic acid, which has an estimated volume of 293 Å^3^ [22].

The other cavity (No. 3) located in the CTD domain is smaller, with a volume of 110 Å^3^. The entrance to this pocket is formed by two negatively charged aspartate residues (D110, D111) but it leads to a rather hydrophobic cavity with the side chains of two valine residues (V154, V170) and of a phenylalanine residue (F168) lining its walls. It might be a binding site for a small hydrophobic ligand with a possible hydrophilic/positively charged terminal group.

Cavity No. 2, located between the NTD and CTD domains, is formed by residues of the loop between α1 and α2, as well as by residues of helix α4 and of strands β5 and β8. The cavity volume is 208 Å^3^ and its interior is hydrophilic, lined by charged amino acid side chains. Although Table 3 lists several hydrophobic residues in this area (V140, F142, L182), only their hydrophilic backbone groups are involved in the formation of this pocket. The only exceptions are two tyrosine residues (Y141, Y181), which could possibly participate in sugar binding. Cavity No. 2 might be, therefore, another putative sugar binding site. In the Bmlp3-p21 structure this pocket is filled by a number of water molecules.

Only one cavity (No. 4) is found to be formed entirely in the NTD (Fig. 6B). It is located between helices α1, α2 and α3, has an elongated shape and is formed mainly by hydrophobic residues. Only two hydrophilic residues (Q13, K29) are present close to its entrance. The calculated volume of this pocket is only 60 Å^3^, however, even a small movement of the helices might enlarge this pocket. Cavity No. 4 could be a fatty acid binding site. This result is intriguing because pocket No. 4 is different from another NTD-located cavity reported for Bmlp7 [22]. The description of the Bmlp7-NTD cavity and its putative fatty acid binding role was reasonable, however, a Bmlp3 counterpart is not detected in our search, even though the NTDs of Bmlp7 and Bmlp3 are very similar.

According to the results presented above, four putative binding pockets can be indicated. However, we would like to emphasize that the indication of potential ligands for a particular site, based on the hydrophilic/hydrophobic character of the cavity, is only tentative and of speculative character.

### Protein Surface

So far, no protein surface analysis has been presented for members of the 30-kDa lipoprotein family. Recently, a study of 30Kc19, an isoform of Bmlp3, has indicated that the protein has cell-penetrating properties [21]. To supplement those results, we have calculated Poisson-Boltzmann electrostatic potential on the molecular surface of Bmlp3 using the APBS algorithm [42] and the PDB2PQR program [43], [44]. As above, chain A of the Bmlp3-p21 structure was used for the analysis. The electrostatic potential was evaluated at pH 7.4, with the corresponding side chain protonation states determined in PropKa [45]. The pH of 7.4 was chosen to correspond to the cell-penetration experiments, which used cell extracts in PBS buffer at pH 7.4 [21].

**Figure 7:**
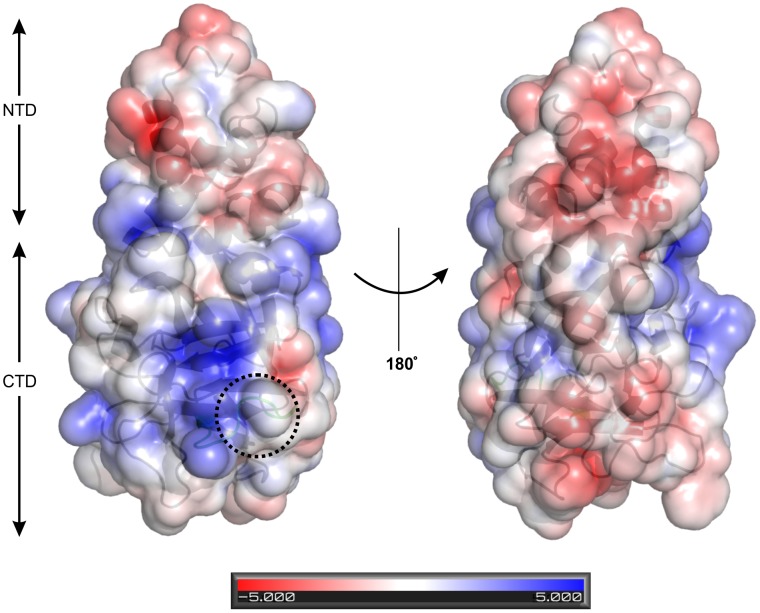
Electrostatic surface potential of Bmlp3 calculated at pH 7.4. Two different views of the surface potential of Bmlp3 are shown. The positive and negative charges are colored blue and red, respectively. The loop containing the putative cell-penetrating signal sequence is marked with a circle.

The surface potential of the NTD domain of Bmlp3 is dominated by negative charges. In the CTD domain positively and negatively charged patches of approximately equal size are located at the opposite sides of the molecule (Fig. 7). This could be interpreted as indicating that the fragments of the CTD domain with positive charged could be involved in receptor-independent endocytosis. This mechanism, described previously for another cell-penetrating protein, HIV-TAT [46], was proposed by Park et al. as an explanation of the cell-penetrating properties of 30Kc19 [21]. Briefly, receptor-independent endocytosis is an energy-dependent process initiated by positively charged amino acid side chains which interact with heparan sulfate in cell membrane. Subsequently, the plasma membrane invaginates and the interacting protein is internalized into the cell. This type of endocytosis is known as macropinocytosis [46], [47], [48]. The amino acid sequence responsible for the cell-penetrating properties of HIV-TAT was established as YGRKKRRQRRR [49]. Interestingly, a similar motif could be found in the Bmlp3 sequence, _116_ YGDGKDKTSPR_126_. These residues form a loop between the β3 and β4 strands. The loop is exposed on the surface of the protein, so it is plausible that it might interact with sulfate moieties in the cell membrane. The loop sequence is the only Bmlp3 sequence fragment consistent with the cell-penetrating motif and is localized in the positively charged area of the Bmlp3 molecule. This observation is a starting point in the elucidation of the cell-penetrating mechanism of Bmlp3. However, it requires further *in vivo* studies.

### Conclusions

The study reports two crystal structures of Bmlp3, a protein belonging to the 30-kDa lipoprotein family found in silkworm hemolymph. So far, only one member of this family, Bmlp7, has been characterized structurally [18], [22]; therefore the presented Bmlp3 structures significantly supplement the existing structural data about insect hemolymph lipoproteins. The protein has two clearly defined domains, a helix-bundle N-terminal domain and a β-trefoil C-terminal domain. A comparison of the Bmlp3 and Bmlp7 structures indicates a high degree of flexibility of the loops present in the CTD.

Four cavities formed in the Bmlp3 structure have been identified and described in detail. Additional experiments aimed at potential Bmlp3-ligand complexes are necessary to shed more light on the ligand binding properties of this protein.

Analysis of the electrostatic potential on the Bmlp3 surface has been used to support the cell-penetrating (macropinocytosis) properties of the 30Kc19 homolog [21]. In particular, a conserved sequence signature identified on a positively-charged patch on the surface of the CTD domain could function as a docking site for cell-membrane heparan sulfates during macropinocytosis.
